# Pancreatic neuroendocrine tumor, lymphoma, and squamous cell carcinoma of hypopharynx; A case report of three primary cancers in one patient

**DOI:** 10.1016/j.ijscr.2019.10.073

**Published:** 2019-11-02

**Authors:** Vorapatu Tangsirapat, Kitti Wongta, Kobkool Chakrapan Na Ayudhya, Vichack Chakrapan Na Ayudhya, Paiboon Sookpotarom

**Affiliations:** Department of Surgery, Panyananthaphikkhu Chonprathan Medical Center, Srinakharinwirot University, Nonthaburi, 11120, Thailand

**Keywords:** Pancreas, Hypopharynx, Neuroendocrine tumor, Lymphoma, Squamous cell carcinoma, Case report

## Abstract

**Background:**

Pancreatic neuroendocrine tumors (PNETs) are rarely found and the majority of PNETs are nonfunctioning. At present, a patient with pancreatic neuroendocrine tumor (PNET), lymphoma, and squamous cell carcinoma (SCC) occurring simultaneously has not been reported. We presented all these three primary cancers in a same patient.

**Case presentation:**

A 51-year-old male presented with a right testicular mass for four months. He underwent right orchidectomy, and the patient was diagnosed malignant diffuse large B-cell lymphoma. Consequently, CHOP regimen and intrathecal methotrexate were given as treatment. During the metastasis work up, a hypodense lesion at the tail of pancreas was found. Distal pancreatectomy and splenectomy were completed, and the pathological report revealed PNET. Eighteen months later, the patient developed a symptom of odynophagia, direct laryngoscopy was performed and a mass at the left arytenoid was found. The biopsy was done and confirmed the diagnosis of SCC. The patient went through concurrent chemo-radiation with a complete response. Currently the patient remains in a complete remission of all three cancers.

**Discussion:**

As the patient had non-functioning PNET and was symptom-free, the diagnosis would have not been discovered without the metastasis workup of lymphoma. Although non-functioning PNET less than 2 cm can be safely observed, the tumor was 4.3 × 1.3 cm and adenocarcinoma was suspected, so the patient underwent the surgery.

**Conclusion:**

Although many cases with multiple primary cancers have been published, a case of patient with PNET, lymphoma, and SCC occurring as primary cancers has not been yet reported.

## Highlights

• We presented a patient with PNET, lymphoma, and SCC occurring simultaneously.
• Malignant lymphoma in this case presented with a slow-growing mass at right testis.
• Hypodense lesion at tail of pancreas by CT scan is rather found in adenocarcinoma.

## 1 Introduction

Neuroendocrine tumors (NETs) are malignant tumors mostly found in the lung, gastrointestinal tract, and pancreas [1]. Pancreatic neuroendocrine tumors (PNETs) constitute the second most common of pancreatic tumors which are accounting for 2.84% with a good prognosis [2]. Most of the PNETs are nonfunctioning and incidentally found on imaging. There have been many reported cases of NETs with various second primary tumors, including lymphoma and squamous cell carcinoma (SCC) [1]; however, up to this date, there has not been yet a report of pancreatic neuroendocrine tumor (PNET) presented with second and third primary cancer. We present a case of PNET in which lymphoma and SCC of hypopharynx simultaneously, and consequently occurred as second and third primary tumor.

This work is compliant with the SCARE checklist and has been reported in line with the SCARE criteria [3].

## 2 Presentation of case

**Fig. 1:**
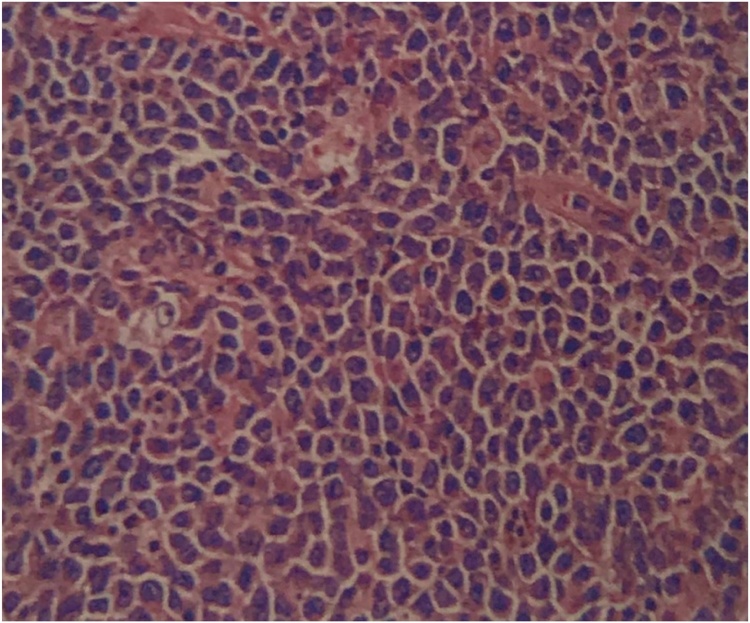
Serial sections of right testis shows diffusely infiltrate of monotonous neoplastic cells, having large nuclei and moderate cytoplasm.

**Fig. 2:**
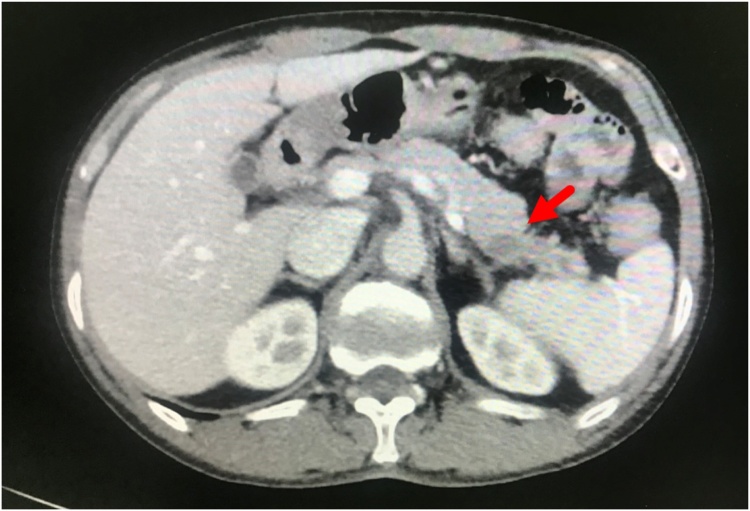
Axial view of contrasted abdominal CT showed irregular hypodense lesion at the tail of pancreas (red arrow).

**Fig. 3:**
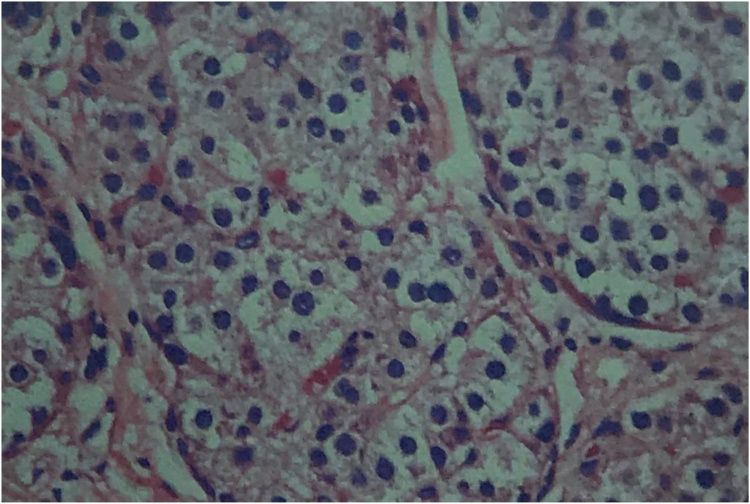
Serial section of tail of pancreas shows multiple clusters and groups of neoplastic cells, having insular formation and infiltrate in the pancreatic parenchymal tissue. Immunohistochemical studies showed tumor cells reactive for neuron-specific enolase and synaptophysin, but non-reactive for CK7 and CK19 antigen.

**Fig. 4:**
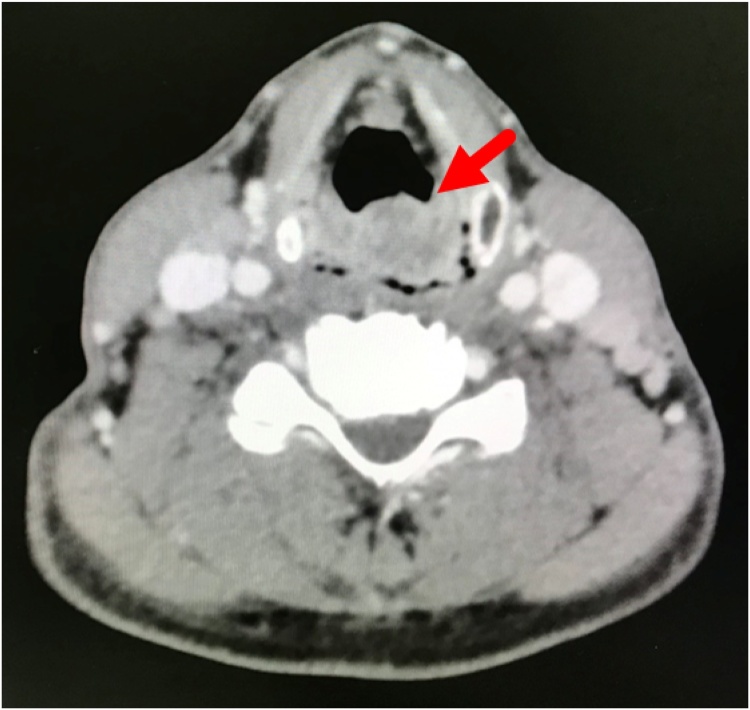
Axial view of contrasted CT showed heterogenous irregular dense mass at anterior wall of hypopharynx with extension anterior to both arytenoid cartilage, posterior commissure, posterior aspect of true vocal cords, subglottic, and pyriform sinuses (red arrow).

A 51-year-old male with a history of hypertension, diabetes mellitus, and smoking presented at our out-patient clinic with a painless, slow-growing mass measuring approximately 15 × 5 cm at right testis for four months. Following advisement and receiving consent, he underwent right orchidectomy. Subsequently, pathologic examination showed malignant lymphoma (Fig. 1) with positive for leukocyte common antigen and CD20 antigen, 90% of Ki-67 antigen expression, but negative for CD3 antigen. As a result, a diagnosis of malignant diffuse large B-cell lymphoma (DLBCL) was made. Computed tomography (CT) scan was obtained and revealed enlarged lymph nodes at right axilla, bilateral upper and lower paratracheal, subaortic, aortocaval, and bilateral inguinal regions. The patient was diagnosed with stage IV DLBCL and was therefore treated with the CHOP regimen and intrathecal methotrexate. During the metastasis work up, the incidental finding is a 4.3 × 1.3 cm irregular, hypodense lesion at the tail of the pancreas was discovered unexpectedly (Fig. 2). Due to the patient was symptom-free, nonfunctional PNET or pancreatic adenocarcinoma was then suspected. The patient had been advised and informed again prior to distal pancreatectomy and splenectomy, and the operation was completed without a complication. The pathology report revealed that the tissue was consistent with PNET grade II. The surgical margins are negative. Immunohistochemical studies showed tumor cells reactive for neuron-specific enolase and synaptophysin, but non-reactive for CK7 and CK19 antigen (Fig. 3). The patient did not receive any adjuvant treatment and was disease-free. Eighteen months after the surgery, the patient encountered problems of odynophagia and occasional aspiration. Thus, direct laryngoscopy was performed and an exophytic mass was found mainly at the left arytenoid and partially involved the right arytenoid. Fortunately, there was no airway compromise. Consequently, tissue biopsy was performed, and the pathological examination confirmed the diagnosis of SCC. CT scan revealed heterogeneous, irregular dense mass at the anterior wall of hypopharynx with anterior extension to both arytenoid cartilages, posterior commissure, posterior aspect of true vocal cords, subglottis, and pyriform sinuses (Fig. 4). The patient went through concurrent chemo-radiation therapy. Subsequently, fiber optic laryngoscopy showed neither masses nor ulcers after the treatment cessation. Currently, one year after the last treatment of SCC, the patient remains in a complete remission of all three cancers.

## 3 Discussion

NETs, tumors arising from hormone-synthesizing cells and cells of the nervous system, are malignant tumors that arise in most epithelial organs of the body. They can be found 0.5% of newly diagnosed tumors and are found mostly in the lung, gastrointestinal tract, and pancreas [1]. PNETs, the second most common of pancreatic tumors, are a subgroup of NETs that originate from endocrine cells of the pancreas [2]. They are categorized into two groups, functioning, and non-functioning PNETs. Patients with functioning PNETs, e.g., insulinoma, gastrinoma, glucagonoma, and VIPoma, would present different symptoms according to different cell types of the tumors. Unfortunately, 70% of PNETs are non-functioning, so the patients are mostly symptom-free [4]. Non-specific symptoms can include abdominal pain, jaundice, pancreatic insufficiency, palpable mass, and anorexia [5]. Consequently, most PNETs are detected by imaging such as CT scan, magnetic resonance imaging (MRI), ultrasound, or positron-emission tomography scan. Although the characteristics of PNETs on CT scan and MRI are hypervascularity in arterial phase and contrast is washed out in venous and delayed phase [6], in the case, since the patient did not undergo triple-phase CT scan of pancreas, only hypodense lesion was found at tail of the pancreas without the information regarding enhancement in the arterial phase. Pancreatic adenocarcinoma was included in differential diagnosis at that time because it is more commonly found. As in this case, the patient had non-functioning PNET and was symptom-free, the diagnosis would have not been discovered without the metastasis workup of the lymphoma as previously mentioned. The curative treatment for PNETs is a removal of the tumor and regional lymph nodes; however, non-functioning tumors less than 2 cm can be safely observed with imaging follow-up [4,5,7]. In our patient, the size of the tumor was larger than 2 cm and pancreatic adenocarcinoma was also suspected, so the patient underwent the surgery. Fortunately, the operation was successful, and the patient recovered uneventfully.

Due to the presence of three cancers in this patient, we try to find whether there are some associations or common predisposing factors in this patient. The only risk factor of PNETs seems to be a genetic factor found in patients with family history of NETs or those with certain familial syndromes such as multiple endocrine neoplasia type I (MEN1), von Hippel-Lindau, tuberculous sclerosis, and neurofibromatosis type I. Nonetheless, the patients in this group usually develop tumor at early age [8]. There are many studies have identified numerous risk factors of DLBCL. These include B-cell-activating autoimmune disease, hepatitis C infection, first-degree family history of non-Hodgkin lymphoma, and young adult body mass index over 30 kg/m^2^ [9,10]. Whereas risk factors of patients with head and neck cancers include smoking, alcoholic drinking, human papillomavirus infection, and exposure to chlorinated solvent [[11], [12], [13]]. In addition, elderly patients with age over 60 years, patients with hypopharyngeal cancers, or heavy drinkers with over 3 drinks per day were also found to be associated with the development of second primary cancers in patients with head and neck squamous cell carcinoma [14]. In our patient, it seems that the only common risk of these cancers might be smoking. Although a genetic study to determine whether there is a trait of certain familial syndromes was not investigated, the association between NETs and second primary malignancies (SPMs) currently is still unknown. In a study analyzing genetic alteration in patients with PNETs, there was a presence of mutation of MEN1, DAXX/ATRX complex, and mTOR pathway genes [15,16]. And there were also abnormalities in the TP53 and retinoblastoma pathway [17]. Interestingly, TP53 genomic defect was found in patients with Li-Fraumeni syndrome, an autosomal dominant syndrome that associated with cancers of breast, soft tissue, brain, adrenal glands, bones, leukemia and lymphoma. The estimated risk of all hematologic malignancies in Li-Fraumeni syndrome is approximately 5% [[18], [19], [20]]. In a genome-wide analysis of head and neck SCC, the authors revealed that there were some genomic alterations. These included HPV and TP53 mutation and smoking and alcohol-related acquired uniparental disomy genomic alteration [21]. As previously mentioned, the patient was not a subject to be genetically investigated. However, TP53 mutation might explain how these primary cancers occurred simultaneously in this patient.

Maybe, a plausible explanation needs an investigation of common carcinogens or causes at the level of stem cells. In a review of studies, the authors found that the association of NETs with SPMs might be due to the common carcinogenic effects or stem cells. Another explanation might be due to the neuroendocrine cell tumors produce the secretory peptides that stimulate the tissue growth that transform into tumors [1,22].

There was a report of 78 patients with NETs of the gastrointestinal tract with SPMs [1]. Of these, SPMs were lymphoma 6.8% and SCC 4.5%. There were only two cases of PNETs in the series, one was found with a neuroendocrine tumor and another was found with an unclassifiable carcinoma as SPMs. There were reports of multiple primary cancers in the literature [[23], [24], [25], [26]]; however, no one had PNET with the accompanying lymphoma and SCC of hypopharynx as the second and third primary malignancies as presented in this case.

## 4 Conclusion

Although many cases with multiple primary cancers have been previously reported, a case of a patient with PNET, lymphoma, and SCC have not been yet published. Moreover, following all treatments of the three cancers, the patient currently is still alive and disease-free.

## Sources of funding

This work received no funding.

## Ethical approval

The consent form and information sheet using in the process of obtaining a consent were approved by IRB at our institution.

## Consent

The patient has been informed prior to the conduction of this manuscript and informed consent has also been obtained. A copy of the written consent is available for review by the editor-in-chief of the journal on request.

## Author’s contribution

Vorapatu Tangsirapat collected data and wrote manuscript.

Kitti Wongta, Kobkool Chakrapan Na Ayudhya, and Vichack Chakrapan Na Ayudhya contributed to conceptualization.

Paiboon Sookpotarom contributed to conceptualization, data curation, supervision and final editing of the manuscript.

## Registration of research studies

N/A.

## Guarantor

Paiboon Sookpotarom.

## Provenance and peer review

Not commissioned, externally peer-reviewed.

## Declaration of Competing Interest

The authors declare that there is no conflict of interest regarding the publication of this article.
